# Supplementary material for: A UV-Protective Textile Coating Based on Recycled Poly(vinyl butyral) (PVB): A New Life for a Waste Polymer
Source: Polymers (Basel). 2024 Dec 7;16(23):3439. doi: 10.3390/polym16233439 (PMC11644265; doi:10.3390/polym16233439)
Supplement: Supplementary file 1 [file polymers-16-03439-s001.zip › polymers-3318454-supplementary.pdf]

A UV-Protective Textile Coating Based on Recycled Poly(vinyl butyral) (PVB): A New Life for a Waste Polymer

Noemi Cei [1], Ilaria Canesi [1,*], Stefano Nejrotti [2,3], Giorgia Montalbano [4], Hamideh Darjazi [4,5], Alessandro Piovano [4,5,*], Matteo Bonomo [2,5], Alberto Fina [6], Beatriz Yecora [7], Angelica Perez [7], Claudia Barolo [2,3], Claudio Gerbaldi [4,5], and Daniele Spinelli [1]

1 Next Technology Tecnotessile, Via del Gelso 13, 59100 Prato, Italy; noemi.cei@tecnotex.it (N.C.); daniele.spinelli@tecnotex.it (D.S.)
2 Department of Chemistry, NIS Interdepartmental Center and INSTM Ref. Centre, University of Torino, 10125 Turin, Italy; stefano.nejrotti@unito.it (S.N.); matteo.bonomo@unito.it (M.B.); claudia.barolo@unito.it (C.B.)
3 Istituto di Scienza, Tecnologia e Sostenibilità per lo Sviluppo dei Materiali Ceramici (ISSMC-CNR), Via Granarolo 64, 48018 Faenza, Italy
4 Department of Applied Science and Technology (DISAT), Politecnico di Torino, Corso Duca Degli Abruzzi 24, 10129 Turin, Italy; giorgia.montalbano@polito.it (G.M.); hamideh.darjazi@polito.it (H.D.); claudio.gerbaldi@polito.it (C.G.)
5 National Reference Centre for Electrochemical Energy Storage (GISEL) – INSTM, Via G. Giusti 9, 50121 Florence, Italy
6 Department of Applied Science and Technology (DISAT), Politecnico di Torino, Viale Teresa Michel 5, 15121 Alessandria, Italy; alberto.fina@polito.it
7 LUREDERRA Technological Centre, Perguita Industrial Area, 31210 Los Arcos, Spain; beatriz.yecora@lurederra.es (B.Y.); angelica.perez@lurederra.es (A.P.)
* Correspondence: ilaria.canesi@tecnotex.it (I.C.); alessandro_piovano@polito.it (A.P.)

Supporting Information

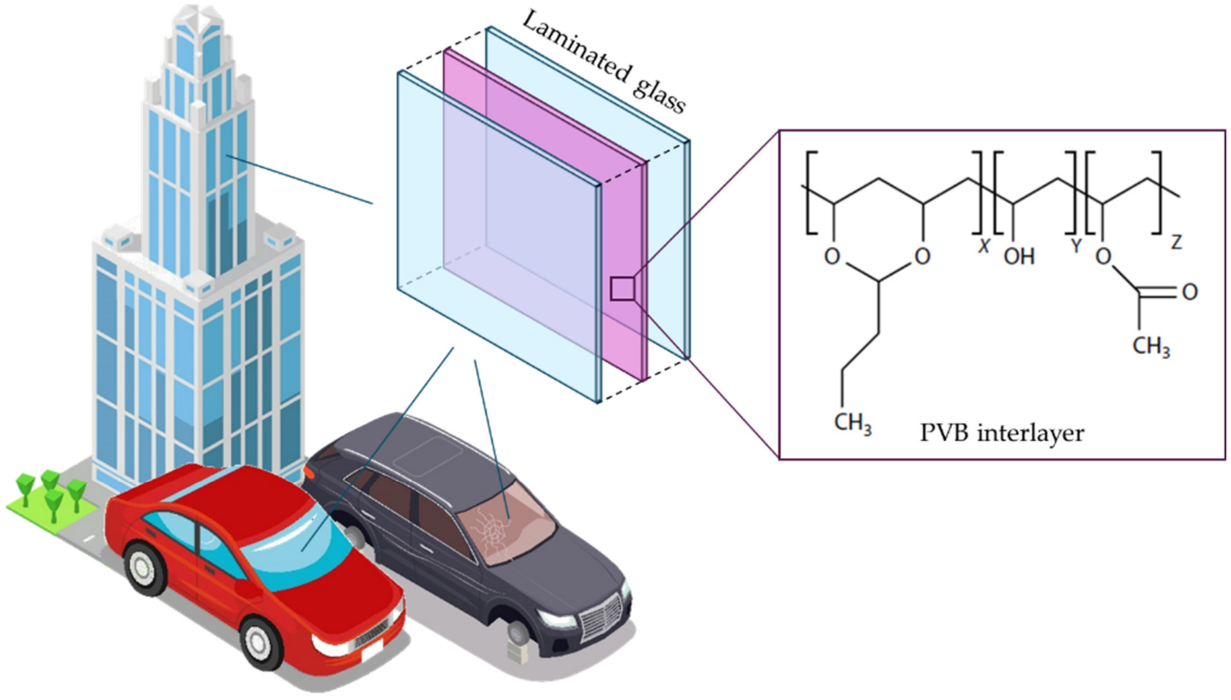

Figure S1. Schematic structure of a laminated safety glass, containing a PVB interlayer (x = vinyl butyral, VB; y = vinyl alcohol, VA; z = vinyl acetate, VAc).

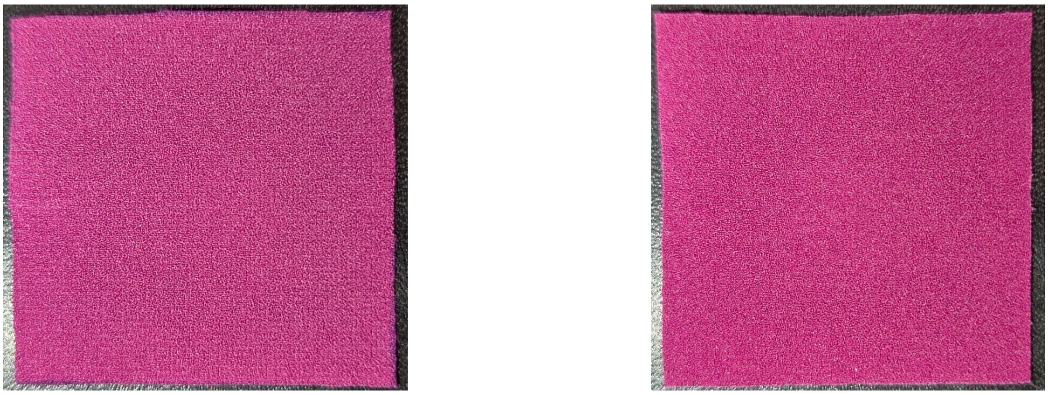

(**a**) (**b**)

Figure S2. Photos of the uncoated (a) and the re-PVB_1-coated fabrics.

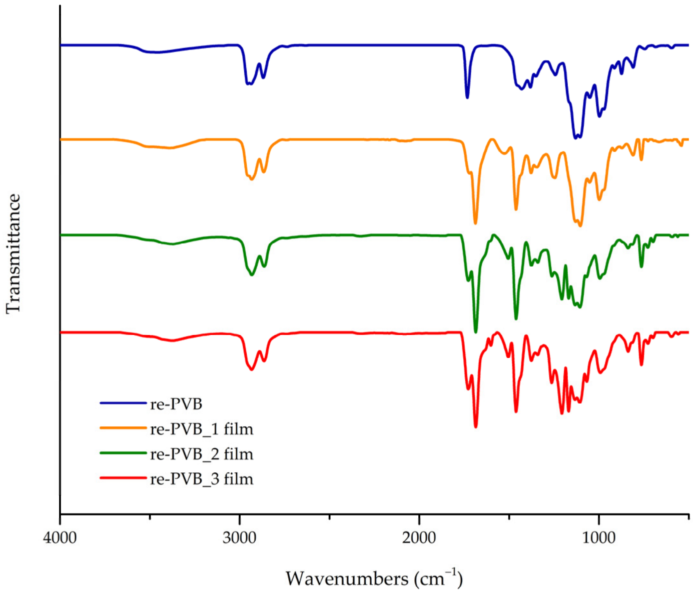

Figure S3. FT-IR spectra of the re-PVB_2 (green) and re-PVB_3 (red) crosslinked film, compared to re-PVB (blue) and re-PVB_1 crosslinked film (orange).

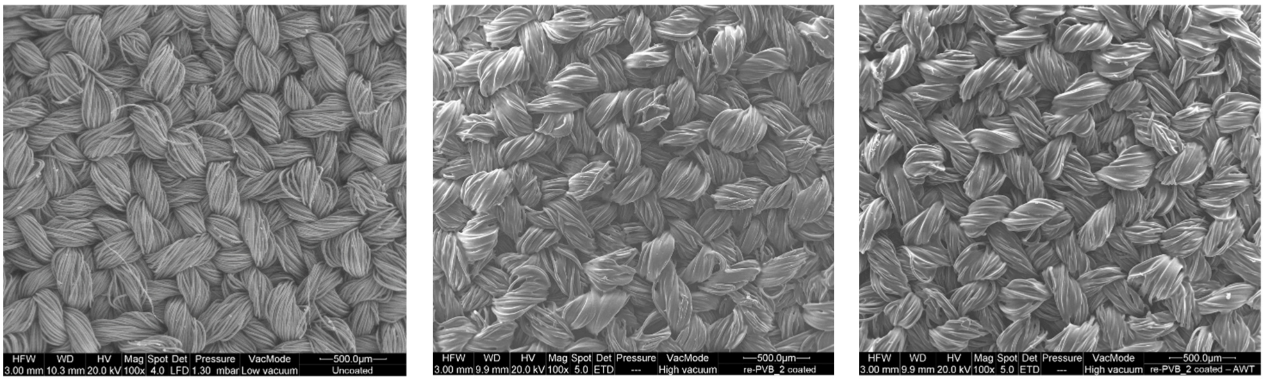

(**a**) (**b**) (**c**)

Figure S4. (**a**) SEM image of the uncoated sample; (**b**) SEM image of the re-PVB_2-coated sample before the washing test; (**c**) SEM image of the re-PVB_2-coated sample after the washing test (AWT).

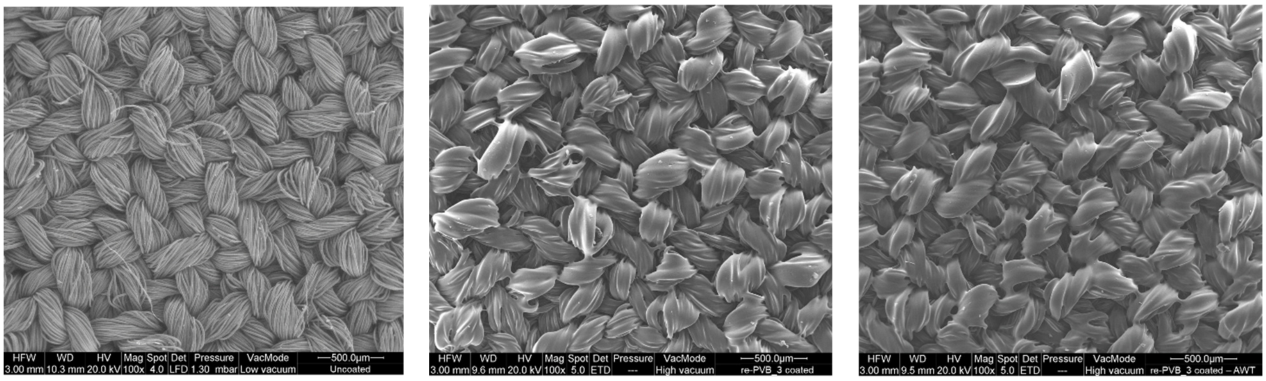

(**a**) (**b**) (**c**)

Figure S5. (**a**) SEM image of the uncoated sample; (**b**) SEM image of the re-PVB_3-coated sample before the washing test; (**c**) SEM image of the re-PVB_3-coated sample after the washing test (AWT).

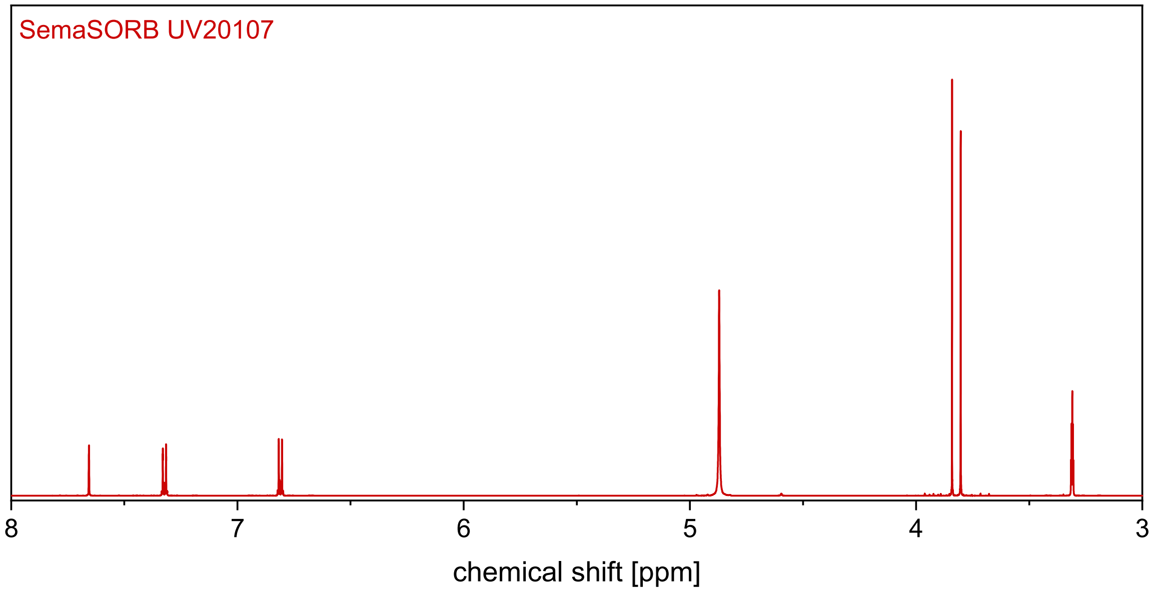

Figure S6. ^{1}H NMR spectrum of SemaSORB® UV20107 in CD_3OD.

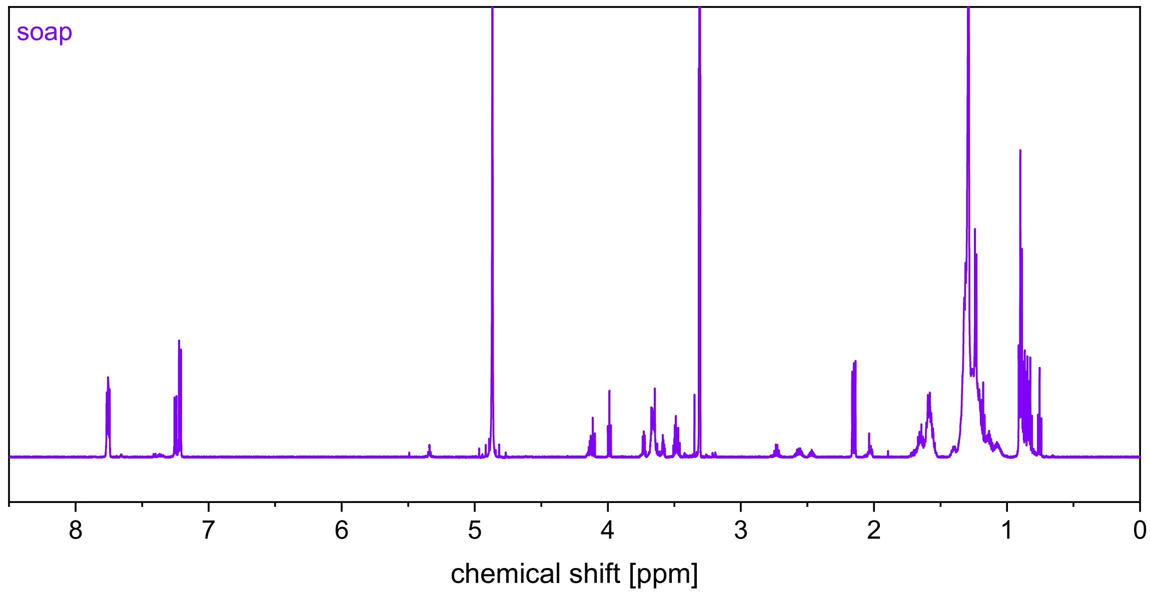

Figure S7. ^{1}H NMR spectrum of the washing soap (*i.e.*, liquid Marseille soap) in CD_3OD solution.

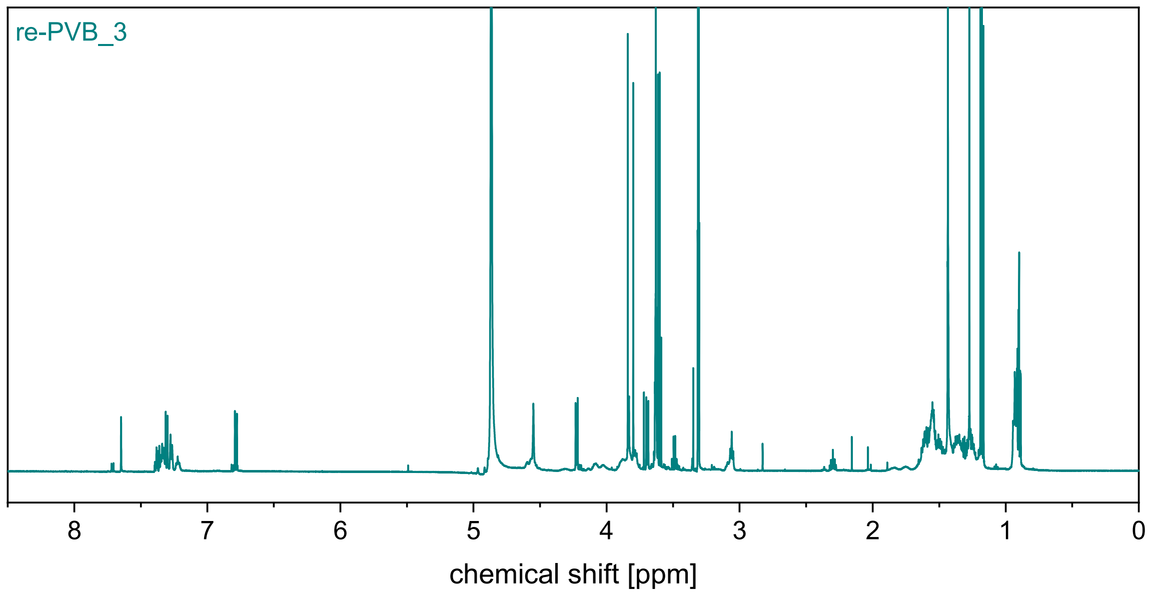

Figure S8. ^{1}H NMR spectrum of re-PVB_3 in CD_3OD solution.

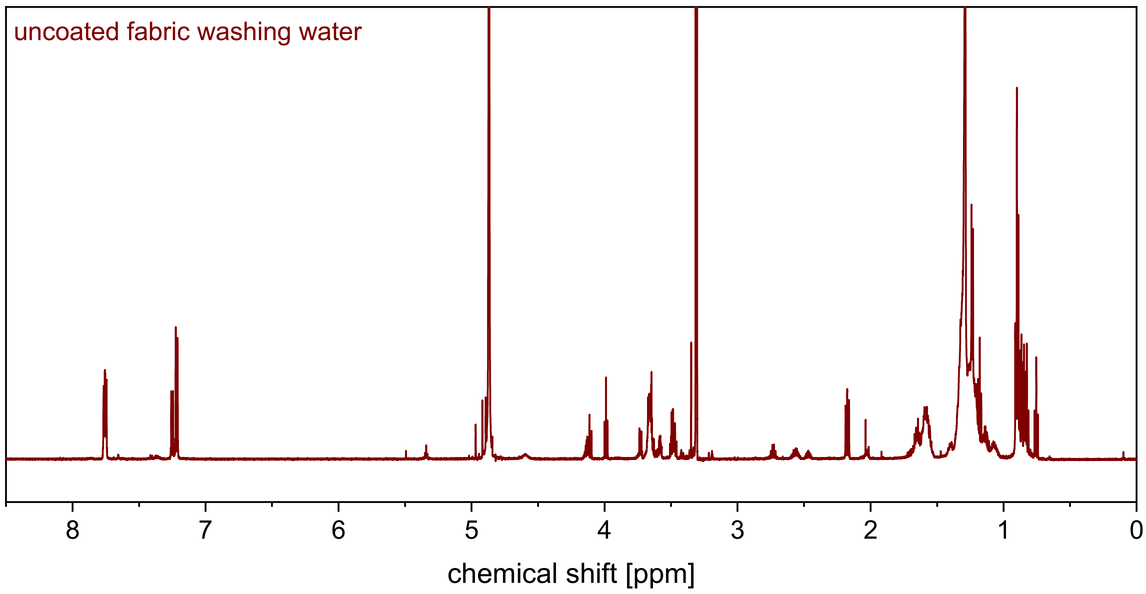

Figure S9. ^1H NMR spectrum of the washing water used for uncoated fabric in CD_3OD solution.

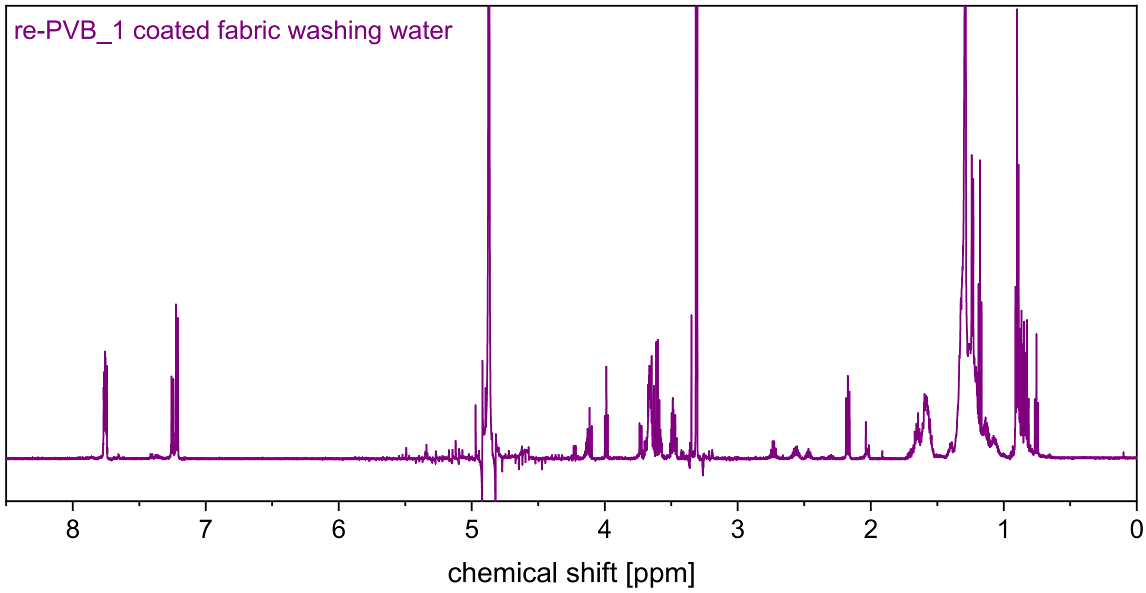

Figure S10. ^1H NMR spectrum of the washing water used for re-PVB_1-coated fabric in CD_3OD solution.

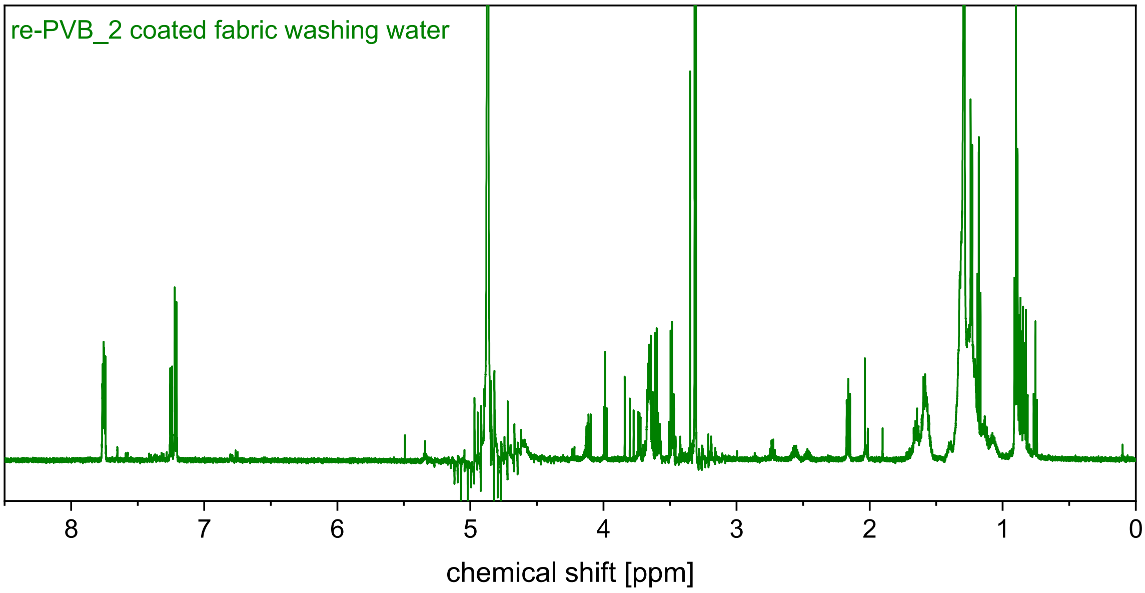

Figure S11. ^1H NMR spectrum of the washing water used for re-PVB_2-coated fabric in CD_3OD solution.

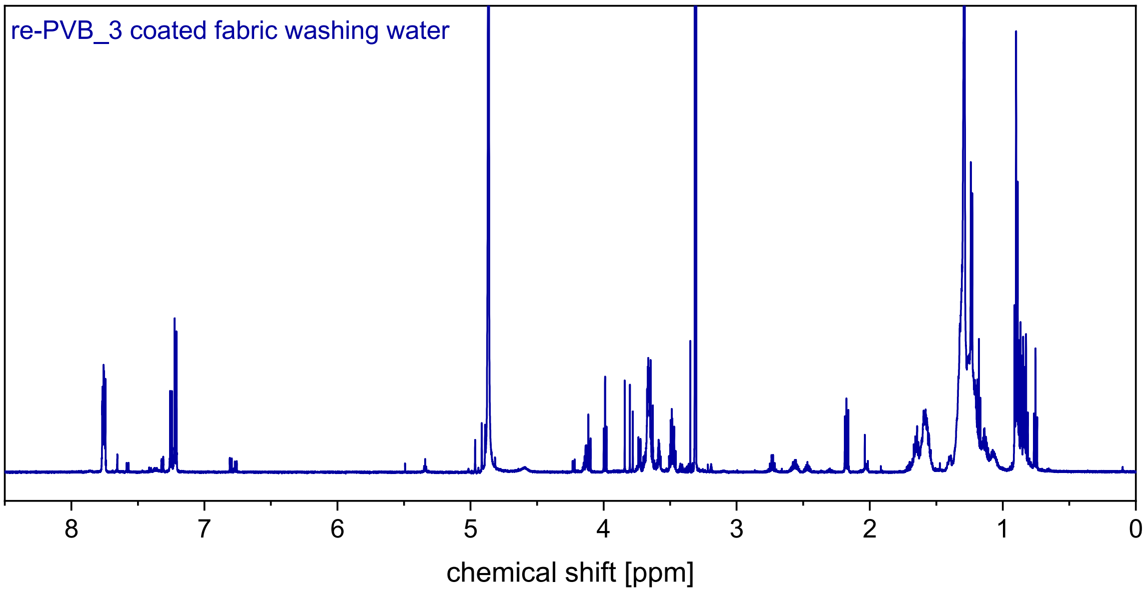

Figure S12. ^1H NMR spectrum of the washing water used for re-PVB_3-coated fabric in CD_3OD solution.

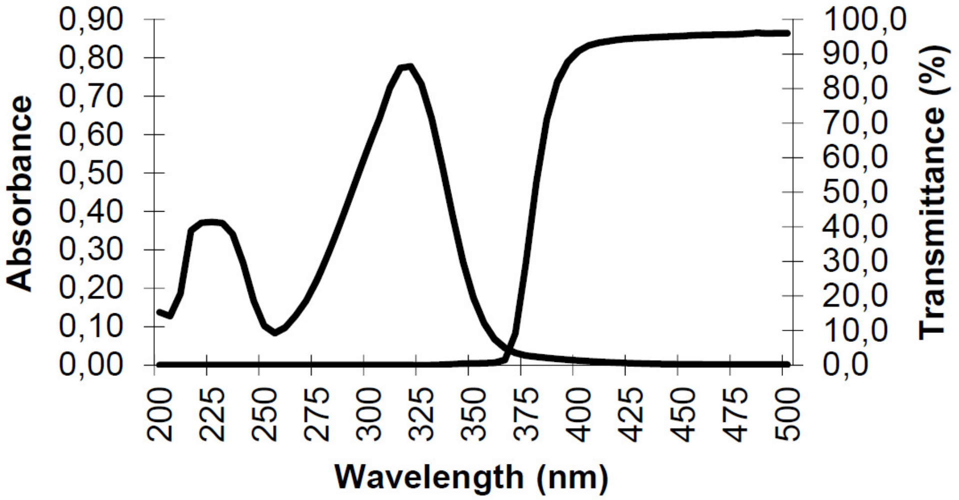

Figure S13. UV spectrum of the UV-absorber SemaSORB® UV20107. Absorbance: 10 mg/l (EtOH); transmittance: 1 g/l (EtOH). [Source: technical data sheet]
